# Evolving dynamic networks: An underlying mechanism of drug resistance in epilepsy?

**DOI:** 10.1016/j.yebeh.2019.03.003

**Published:** 2019-05

**Authors:** Wessel Woldman, Mark J. Cook, John R. Terry

**Affiliations:** a Living Systems Institute, Centre for Biomedical Modelling and Analysis, EPSRC Centre for Predictive Modelling in Healthcare, University of Exeter, United Kingdom; b Department of Medicine – St. Vincent's Hospital, The University of Melbourne, Parkville, VIC 3010, Australia; c Graeme Clark Institute, The University of Melbourne, Parkville, VIC 3010, Australia

**Keywords:** Anti-epileptic drugs (AEDs), Drug tolerance, Drug-resistant epilepsy (DRE), Computational model, Prognosis

## Abstract

At least one-third of all people with epilepsy have seizures that remain poorly controlled despite an increasing number of available anti-epileptic drugs (AEDs). Often, there is an initial good response to a newly introduced AED, which may last up to months, eventually followed by the return of seizures thought to be due to the development of tolerance. We introduce a framework within which the interplay between AED response and brain networks can be explored to understand the development of tolerance. We use a computer model for seizure generation in the context of dynamic networks, which allows us to generate an ‘in silico’ electroencephalogram (EEG). This allows us to study the effect of changes in excitability network structure and intrinsic model properties on the overall seizure likelihood. Within this framework, tolerance to AEDs – return of seizure-like activity – may occur in 3 different scenarios: 1) the efficacy of the drug diminishes while the brain network remains relatively constant; 2) the efficacy of the drug remains constant, but connections between brain regions change; 3) the efficacy of the drug remains constant, but the intrinsic excitability within brain regions varies dynamically. We argue that these latter scenarios may contribute to a deeper understanding of how drug resistance to AEDs may occur.

## 1 Introduction

Epilepsy is a serious brain disorder with a lifetime incidence of around 1%. One of the many challenges for neurologists treating epilepsy is that at least one-third of all patients have seizures that remain poorly controlled despite the increasing number of anti-epileptic drugs (AEDs) now available, so-called drug-resistant epilepsy (DRE). This number has remained frustratingly static, with no significant improvement over the past 30 years [1]. An intriguing phenomenon is termed tolerance, or the ‘honeymoon effect’, where after an initial good response to a newly introduced medication, seizures return to the previous frequency after a period of weeks or months. The phenomenon is most often described with benzodiazepines but is recognized to affect most, if not all, AEDs [2], [3], [4]. Several mechanisms have been proposed to explain this phenomenon, including metabolic (pharmacokinetic) tolerance, due to induction of AED-metabolizing enzymes or blood–brain-barrier multidrug transporter proteins, and functional (pharmacodynamic) tolerance, related to alteration of AED targets through loss of receptor sensitivity or similar mechanisms [4], [5]. There is a good deal of evidence, both clinical and experimental, regarding these mechanisms, but none satisfactorily explains clinical observations regarding the development of tolerance. Viewing the concept of tolerance more holistically, in particular to incorporate disease-related mechanisms, may enable us to reconcile these common clinical observations [3]. Towards this aim, we consider the role that neural plasticity, particularly at the level of large-scale brain networks, might play.

In recent years, our understanding of the role that the topology of large-scale brain networks plays in shaping neuropathology has greatly advanced [6]. In the case of epilepsy, it has been shown both conceptually and using clinical EEG in the case of generalized seizures that perturbations to large-scale network structures, or to dynamics within localized brain regions, can both lead to seizure activity [7], [8]. In this brief communication, we consider conceptually the interplay between AED effects, brain network structures, and localized dynamics to describe candidate mechanisms by which tolerance could emerge over a timescale of months.

## 2 Methods

A dynamic network model was used to simulate networks of six interconnected brain regions with asymmetric directed connections between them. The model is constructed as follows. Our starting point is an equation previously introduced in the context of epilepsy [9]:

(1) $$z^{\prime }=f\left(z\right)+\mathrm{\alpha d}w^{\prime }=\left(\lambda -1+\mathrm{i\omega}\right)z+2z\left|z^{2}\right|-z\left|z^{4}\right|+\mathrm{\alpha d}w^{\prime }$$

This is a stochastic differential equation and is used to describe the dynamics within a single brain region. Such an equation describes the evolution over time of a variable *z*, whose output may be thought of as a proxy for the electrographic activity recorded within a single channel of EEG overlying that brain region. This equation was chosen as it allows two different types of dynamic behavior to simultaneously coexist. One is a low-amplitude steady-state solution (that we consider a characteristic of interictal brain states). The other is a high-amplitude oscillatory state (characteristic of ictal brain activity) [10]. This simplified description serves as a proxy for the brain as it transitions from nonseizure to seizure states and back again.

**Fig. 1:**
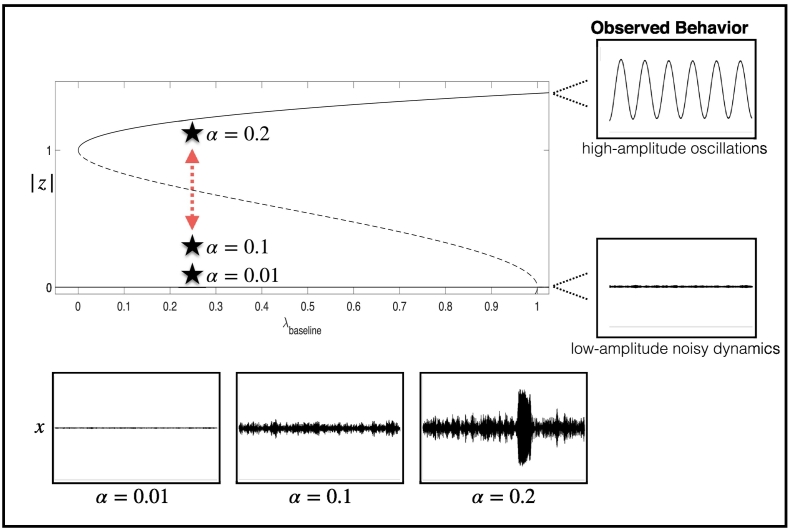
Illustrating the different types of behavior of the system. Dynamics of the system will naturally settle into the low-amplitude state (bottom right representing interictal dynamics, which is defined by the term (*λ* − 1 + *iω*)*z* in the equation) or the high-amplitude oscillatory state (top right representing ictal dynamics, which is defined by the term − z | z |^4^ in the equation). However, remaining in the same state is critically dependent on perturbations from the noise term being relatively small. If we assume we are initially in the low-amplitude (interictal) state, the system will be slightly perturbed for small noise (bottom left). However, for increasing values of the noise term α (reflected by 0.01, 0.1, and 0.2), eventually, the amplitude of noisy perturbations received by the system is strong enough to drive the behavior into the high-amplitude oscillatory (ictal) state. The dotted line in the figure effectively defines a boundary between the two different states: only if the noise perturbation is large enough to cross this boundary can a seizure to occur in our model (this is defined by the term 2*z* |* z* |^2^ in the equation).

The parameter *ω* determines the frequency of oscillation. An appropriate choice ensures that the output oscillates around three cycles per second (similar to the frequency seen during seizure activity). *dw* is a noise term that has an amplitude defined by *α*. Depending on the size of the perturbation delivered by the noise term at each instance in time, it is possible to transition from one dynamic state (e.g., the interictal state) to the other (e.g., the seizure-like state), see Fig. 1. This noise term is included to account for the multitude of dynamic inputs each brain region will receive that are not captured explicitly by the dynamic Eq. (1).

The variable *λ* effectively controls the likelihood of transitioning between the interictal and ictal states within a brain region, effectively how ‘excitable’ each brain region is, and is defined by the following ordinary differential equation:

$${\tau \lambda }^{\prime }=\lambda_{\mathrm{baseline}}-\lambda -\left|z\right|-\lambda_{\mathrm{AED}}$$

The overall transition likelihood depends on a fixed parameter for the system *λ*_*baseline*_, which is inhibited by the overall level of excitability *λ*, the activity in the system |* z* |, and the effect of the AED (*λ*_*AED*_). A system with low baseline level of excitability (*λ*_*baseline*_ close to 0) can be thought of as a ‘normal’ brain region whereas a region with *λ*_*baseline*_ close to 1 can be thought of as strongly ictogenic. Note that the effect of the AED is modeled within the range *λ*_*AED*_ ∈ [0, 1], which effectively reduces the excitability of a region, making the transition to ictal activity less likely. In this representation, we do not ascribe any neurobiological mechanism to the reduction in excitability elicited by the AED, rather, we think phenomenologically of the desired effect of an AED being to reduce the level of emergent ‘excitability’ within a brain region. As shown in Fig. 1, the onset of a seizure in this model critically depends on the specific choice of parameters, most notably *λ*_*baseline*_ and *α*. If *λ*_*baseline*_ is close to 0, only a large value for *α* would drive the system towards the ictal state whereas if *λ*_*baseline*_ is close to 1, even small values of α would be enough to drive the system into a seizure. *τ* is a given time constant, which effectively determines the duration of a seizure. Typical values of parameters and variables are presented in Table 1.

**Table 1:** Description of model variables, parameters, and components.

Variable	Description	Dimension
*z*(*t*) = *x*(*t*) + *iy*(*t*)	Complex state variable	2
*λ*(*t*)	Excitability of a brain region	1
*w*(*t*)	Complex Wiener process (a stochastic process known as Brownian Motion)	2


Parameter	Interpretation	Typical values
*ω*	Frequency of limit cycle	20 (rad/s)
*α*	Noise amplitude	0.0535
*τ*	Typical duration of return to excitability—baseline	50 (s)
*λ*_*baseline*_	Baseline level of excitability of a brain region	0.60
*λ*_*AED*_	Effect of AED on the overall excitability of a brain region	0.10
*β*	Coupling strength	0.35
*N*	Number of nodes in the network	6
**A**	Adjacency matrix (*N* × *N*)	[0,1]


Model component	
(*λ* − 1 + *iω*)*z*	Stable fixed point: corresponding to an interictal resting-state
−* z* |* z*^4^ |	Stable fixed point: corresponding to an oscillatory seizure-state
2*z* |* z*^2^ |	Unstable limit cycle: separates the two basins of attraction

It is important to reiterate that this is a purely phenomenological description that aims to describe the theoretical phenomenon of a transition into the seizure state; this means that a model simulation replicates at a conceptual level the fundamental components of the transition into a seizure but does not have the same richness and level of detail as an actual recording of a seizure. This is much in the way that an animal model typically presents a phenotype that may be related to the human condition but is, of course, not a substitute thereof.

We can extend these equations for a single region into a network of coupled regions, where the dynamics of each region would correspond to the activity captured by an EEG channel overlying that region. The consequent “dynamic network model” consists of coupling together N such regions (whose topology or structure is defined by the adjacency matrix **A** – effectively a set of ‘0’s and ‘1’s where ‘1’ indicates a connection between two regions), with the dynamics of region i described by:

$${z_{i}}^{\prime }=\left(f\left(z_{i}\right)+\beta \sum_{j\neq i}^{N}A_{\mathrm{ji}}\left(z_{i}-z_{j}\right)\right)+\mathrm{\alpha d}w_{i}{\left(t\right)}^{\prime }.$$

Here, *f*(*z*_*i*_) is defined as previously. In the context of this phenomenological description, a connection between two regions is “synchronizing”. This means that region i will influence region j to exhibit the same dynamic state that it is in (e.g., interictal or ictal) if there is a connection between regions i and j (due to the term (*z*_*i*_ − *z*_*j*_)). As a conceptual study, we do not attempt to ascribe any underlying neurobiological context (e.g., an excitatory or inhibitory process) to these connections; defined by the matrix **A**, they represent an abstract representation of a brain and are not derived from imaging data (e.g., EEG, magnetoencephalography (MEG), or functional magnetic resonance imaging (fMRI)). Herein, we consider a set of six interconnected brain regions as a pragmatic balance between complexity and ease of illustration.

A critical determinant of level of influence is *β*, the global coupling strength, which determines the overall influence that connected regions within the network can have on each other. Whether a transition to seizure is observed is therefore dependent on the interplay between the level of excitability and the intrinsic noise term within each brain region and the synchronizing effect of other brain regions as a result of the given connections within the network.

## 3 Results

**Fig. 2:**
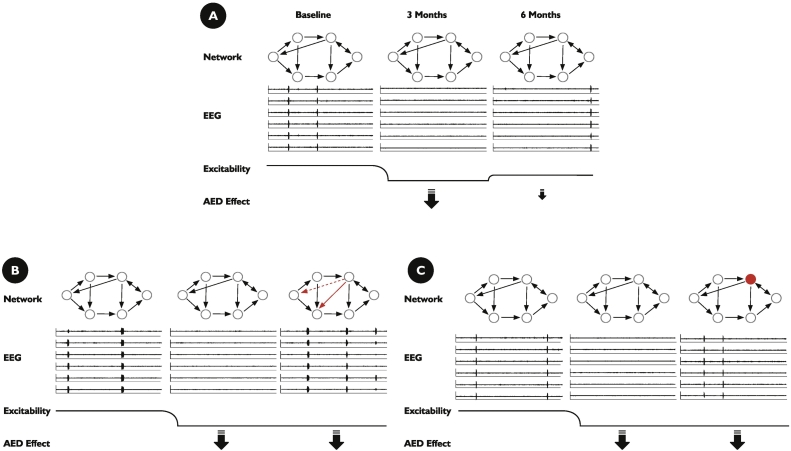
Illustrating 3 scenarios by which tolerance to antiepilepsy drugs might occur. In scenario A, the classical concept of a “honeymoon” period after which a prescribed medication becomes less effective leading to the return of seizures. This corresponds to *λ*_*AED*_ = 0 at baseline, *λ*_*AED*_ = 0.15 at 3 months (the time the new AED is first administered), and decreased to *λ*_*AED*_ = 0.075 at 6 months as the efficacy of the AED diminishes as ‘pharmacological’ tolerance develops, and seizure activity recurs. Scenario 2 considers how an alteration to large-scale brain networks could result in a situation where a drug ceases to be effective as the network has become more “ictogenic” over time and so the same level of AED effect ceases to ensure effective seizure control. In scenario 3, there is an alteration to a localized brain region in such manner that it also increases the overall ictogenicity of a network. Once more, this renders the same level of AED ineffective for seizure control. In scenarios B and C, we have *λ*_*AED*_ = 0 at baseline and have maintained *λ*_*AED*_ = 0.15, the previously effective level, at 3 and 6 months. In scenario B, the network structure changes by altering a single edge within the network, and in scenario C, the excitability of one node in the network is changed. Seizure recurrence then is a consequence of changes in the network structure or dynamics, rather than through pharmacological tolerance developing. See Table 1 for all other choices of the parameters; the system of Stochastic Differential Equation (SDEs) was solved using the Euler–Maruyama method with a time step of 0.0001.

The starting point for each scenario was a six-region network (see Fig. 2), where the probability of interictal to ictal transition was significant (> 1 Sz/week). For simplicity of analysis, parameters defining the brain dynamics within each node were identical, and similarly, the strength of each formed connection was the same (see Table 1 for parameter choices).

### 3.1 Scenario 1

The first scenario we consider is designed to illustrate the classical concept of the ‘honeymoon’ period (Fig. 2A). In this case, the administration of the AED has an effect modeled as a reduction in the overall level of excitability in each region. For the given brain network, the effect of this reduction in excitability is to reduce the probability of a transition from interictal to ictal dynamics to a negligible level. However, over time, the effect on excitability of the AED begins to wane, and levels increase back towards baseline. Beyond a certain point, the probability of a transition to ictal dynamics becomes significant, making observation of a seizure likely.

### 3.2 Scenario 2

In the second scenario, we consider the role that neural plasticity, as reflected in the evolution of large-scale network structures between brain regions, may play (Fig. 2B). Again, we start from a network of brain regions that supports regular transitions from interictal to ictal dynamics. As before, if we consider that the initial administration of the AED diminishes the overall excitability of the network, we see a temporary absence of seizures in the model at 3 months. We then examined the case where at some point between 3 and 6 months, the network structure between brain regions has evolved subtly. In particular, we illustrate the case where a change in a single edge causes the return of ictal dynamics after 6 months.

### 3.3 Scenario 3

In the final scenario, we consider the role that neural plasticity, as reflected in a change in internal dynamics within a brain region, may play (Fig. 2C). In this case, we again start with a brain network that supports regular transitions from interictal to ictal dynamics. In this case, while the effect on overall excitability mediated by the AED remains the same, and there is no alteration to the network structure, over time, the internal excitability within a specific brain region increases, such that the probability of transition from interictal to ictal dynamics becomes significant at six months, making observation of a seizure more likely.

## 4 Discussion

Understanding the mechanisms of drug resistance is a necessary step to develop strategies to mitigate this problem [11], [12]. Characterizing the phenomenon of tolerance to AEDs has classically focused on microscale mechanisms of drug (inter)actions in the brain, assuming that the phenomenon of tolerance is associated with either a pharmacokinetic or a pharmacodynamic effect [11]. Given that the brain is highly plastic, it is therefore likely that epilepsy is an evolving process. Epilepsy is now widely accepted to be a network disorder, and seizure generation critically involves large-scale brain networks [13]. How mechanisms across these different scales interact, and how likely seizures are to occur as a result, remain open questions. Our study provides a different perspective on the development of tolerance, which often leads to DRE, suggesting that this phenomenon may be understood as a manifestation of adaptive network changes [14], [15].

Within this framework, we assume that the action of an AED at the macroscopic level is to reduce the level of excitability within brain regions. We present three scenarios by which tolerance to AEDs can occur. The first assumes that a brain network remains relatively static over time while the efficacy of the drug diminishes. This scenario can be thought of as the classical case: drug efficacy diminishes because of some unknown (presumed microscale) mechanism, and increased excitability of the brain leads to the return of recurrent seizures. However, we further describe two other scenarios where seizures can recur. In both cases, there is no change to the efficacy of the drug, rather, properties of the large-scale brain network evolve dynamically over time. One case explores alterations to connections between brain regions, as we might expect from neuroplasticity [16], [17]. The second considers the scenario where intrinsic excitability within brain regions varies dynamically. This latter scenario may go some way towards explaining the phenomenon of fluctuating response, where a significant minority of people with epilepsy (~ 15%) experience long periods of seizure freedom interspersed with time windows, in the order of months, where seizures are ongoing [1]. It is important to point out that this is not an exhaustive list of potential scenarios, indeed, there could be several others, notably combinations of the scenarios we have considered occurring simultaneously. Ultimately, further studies using neurophysiological and electrophysiological recordings from people with epilepsy who actually experienced the honeymoon effect would allow for robust testing of these scenarios, as well for further research on the underlying mechanisms responsible for the observed honeymoon effect.

Such future studies focused on understanding how functional representations of brain networks (either from EEG or fMRI) vary over time may play a critical role in improving our clinical understanding of the relative contribution of microscopic and macroscopic variables in seizure generation and therefore the overall response to AEDs. For example, long-term EEG monitoring could enable the identification of changes in network structure or model properties related to changes in medication or dosage. To date, recordings of sufficient duration to capture changes of this nature have not been available to study changes in the dynamics of brain activity over the long periods necessary but will likely soon be possible, for example using subscalp electrodes [18], [19]. These studies ultimately may have prognostic value: revealing patient-specific features of drug efficacy that enable an optimal treatment regime.

## Acknowledgments and disclosure of conflict of interest

Wessel Woldman received financial support from the Medical Research Council (via MR/N01524X/1). John R. Terry and Mark Cook received financial support from a Royal Society International Exchanges Award (grant number IE170112). John R. Terry further acknowledges the generous support of the Wellcome Trust Institutional Strategic Support Award (WT105618MA), and the EPSRC (via Centre Grant EP/N014391/1). We have no conflict of interest to declare. We confirm that we have read the Journal's position on issues involved in ethical publication and affirm that this report is consistent with those guidelines. Full Matlab scripts will be made available on an appropriate repository (e.g., GitHub).
